# Infodemic management challenges and evidence-based midwifery

**DOI:** 10.18332/ejm/168728

**Published:** 2023-08-25

**Authors:** Victoria G. Vivilaki, Elisabeth Wilhelm, Elena Petelos

**Affiliations:** 1 Department of Midwifery, School of Health and Care Sciences, University of West Attica, Athens, Greece; 2 Information Futures Lab, School of Public Health, Brown University; 3 Health and Society Laboratory, Faculty of Medicine, University of Crete, Heraklion, Greece; 4 Health Services Research, CAPHRI Care and Public Health Research Institute, Faculty of Health, Medicine and Life Sciences, Maastricht University, Maastricht, The Netherlands

**Keywords:** infodemic management, challenges, evidence-based midwifery

## What is an infodemic and how does it affect evidence-based midwifery?

The World Health Organization (WHO) defines an infodemic as an overwhelming amount of information, including misinformation and disinformation, that can circulate during acute events, making it hard for people to identify trustworthy sources and reliable guidance^1^. However, infodemics can, also, interfere with health-seeking behaviors and evidence-based practice at any time, affecting women and their families across communities, including in the context of seeking routine sexual and reproductive healthcare^2^. Today, midwives can help protect their health by identifying the best possible evidence for new technologies and combating the infodemic in the context of evidence-based midwifery practice.

Most people, and certainly women of child-bearing potential, pregnant women, and young mothers, now, search for health information through mobile devices and through their social networks online and offline, even if they still consider healthcare professionals as their most trusted sources of health information, and midwives as their most trusted partner in the perinatal period. Infodemic management challenges are complex, systemic, and hard to quantify for midwives, because the quality of information, including the level of evidence, available on reproductive health, fertility and pregnancy is heterogeneous. Low quality information and misinformation is readily available and appealing to women of child-bearing potential, which can successfully compete against the evidence-based advice a midwife may provide^3,4^. Identifying the best available evidence may also pose concrete challenges for midwives, with evidence from clinical trials often being extremely limited when it comes to women of child-bearing potential, pregnant women, lactating women, etc.; a key limiting aspect in both in terms of the autonomy of women and of the practice of midwives, as it became starkly clear during the COVID-19 pandemic^5^. Furthermore, midwives need to learn how to navigate an increasingly digitized information environment for themselves and the women they serve, and to help them and other community health actors deepen their engagement with communities.

Given the digital divide experienced across many communities, midwives need to both navigate the infodemic, as experienced during the COVID-19 pandemic and in the post-COVID-19 era, and to bridge this divide along with the women, the families, and the communities they support, picking up new digital and infodemic management skills^6^.

There is, therefore, a need to define and establish the role of midwives in infodemic management and to equip them for it by developing and deploying appropriate, effective and efficient capacity-building strategies and tools. An emerging challenge, for example, relates to ability to utilize digital technology to access, understand and use health information effectively for themselves and their clients. Midwives’ role extends from identifying evidence for evidence-based practice, to dissemination of information, to improving acceptance and adherence as part of health promotion and prevention activities, and for treatment too, and across a wide range of technologies, i.e. vaccines, to digital diagnostics and therapeutics, etc. Because midwives take a relational rather than a transactional approach to community engagement, including specific field actions like postnatal home visiting, they are well suited to using infodemic management approaches effectively and efficiently at the interpersonal and community levels.

## Why is it important to understand how people search and use health information?

Topics that midwives need to address are often poorly understood by lay women, particularly marginalized and vulnerable women groups. The social determinants of health – including access to media and information sources to digital and health literacy – can affect a woman’s ability and access to health information as well^7^. Often, readily available social media and online content on health topics may be more easily found, more engaging, and more attractively packaged than the more fact-based content from trustworthy health information sources. Low-quality or inaccurate social media-optimized content can include stigmatizing messages, promote potentially harmful treatments, and even reinforce harmful gender stereotypes. Algorithms are primed to surface content which gets high engagement with users and viewers, making it harder to find credible information that is not optimized for online engagement.

Midwives can leverage the latest research and infodemic insights to inform their approaches to address health misinformation successfully. Social listening data, which may detect questions, concerns, information voids, narratives and circulating misinformation and disinformation, can offer powerful insights to midwives on how to help their clients navigate their information environment. These insights may highlight trends around common misinformation narratives, specific influencers who may be propagating inaccurate information, online marketing ads of traditional healers or others that are promoting non-evidence-based treatments^8^. They can also uncover common questions or concerns where people are looking for information about a health topic online and cannot find credible sources. Narratives that emerge of common and recurring questions, concerns or misinformation can also be assessed for risk, particularly to vulnerable groups such as pregnant women. High risk narratives may encourage risky health behaviors, promote stigma or affect adherence, resulting in further impact on trust in health interventions and services^9^. For example, the COVID-19 pandemic highlighted the extremely low vaccine uptake among pregnant women, with vaccine safety fears and misinformation cited as main reasons why they were not choosing immunization despite well-documented risk of adverse health outcomes for mother and baby in case of infection – high risk narratives that were widespread globally^10^.

Although a midwife’s role is not to primarily factcheck the Internet, they are uniquely positioned to counteract such potentially harmful content by individualized care when tailoring healthcare to the unique needs, preferences, and circumstances of each woman and her family. Interpersonal communication approaches, long used to improve the quality of conversation between women and midwife, such as motivational interviewing (MI), can be complemented with specific approaches to address misinformation, including providing debunking and unmasking approaches used in science denialism^11,12^. Additionally, midwives who are equipped with digital skills can also play a role in engaging with people and communities online to more effectively share credible, accurate health information and address misinformation^13^.

A midwife’s ability to pair credible health message sharing with empathy and commitment to a woman’s health, in both virtual and physical spaces, is a powerful combination that can serve women well, regardless of where they may be. This allows the midwife to develop a trusting relationship with the woman and her family, understand their individual needs and preferences, and provide personalized support and guidance. Moreover, midwives value informed choice and shared decision-making. They provide information, explain available options, and involve women in decision-making processes related to their care. This empowers women to make choices that align with their values and preferences while ensuring they have a clear understanding of the benefits, risks, and alternatives. Overall, individualized care by midwives acknowledges the unique experiences and needs of each woman and her family. By providing personalized, woman-centered care, midwives promote positive health outcomes, empower women, and enhance the overall childbirth experience.

This is especially true in any situation of rapid social reordering triggered by crisis (economic, pandemic, conflict, etc.) where there may be concerns of loss of trust in governments, and where community midwives that have already established and continue to build strong bonds with the local women are more likely to succeed. Notably, compassionate care delivery may have a role to play in building networks for establishing and utilizing useful narrative to counter misinformation and disinformation^14^. The combination of low trust and crisis can be provide fertile ground for the spread of misinformation, including health misinformation, because people’s typical networks and pathways to healthcare may be affected. It can also affect midwives directly. Extortion and violence against public health professionals, in particular, have become more pervasive than many assume. Health workers who have addressed misinformation online and publicly have experienced harassment and doxing, meaning their personal information is uncovered and posted online for the world to see^15^. Tools to equip midwives to protect themselves and build supporting professional networks are required to meet these new challenges, especially if they are operating in a context where they be promoting important public messages for safe motherhood in a digital environment where it can be unsafe for the midwives who do it.

## Right of access to information and to care for all women and girls

Every woman should have the right to access the health information she needs to ensure their autonomy, to keep herself, her pregnancy and her family safe. Equitable access to healthcare is a longstanding goal for all health systems, but so should the right to equitable access to timely and trustworthy health information stemming from the best available evidence. Reshaping the essential and wonderful role of midwives have to play, and connecting women to care, despite challenges that may stand in the way, necessitate interprofessional collaboration with public health professionals and interdisciplinary research to help shape priorities and generate evidence on all emerging and longstanding challenges. Such challenges range from infrastructure (does the woman have access to information sources or the Internet?), to social (do online and offline community norms support a woman’s healthcare choices?), and to individual (does a woman have the digital and health literacy skills to navigate the mix of information she finds?). In some communities, gender can also play a role in access to information, including negotiating access to a phone or Internet.

Despite these barriers, a midwife can add more tools in her armamentarium to reach pregnant women if she is exposed to new infodemic management approaches to address these 21st century barriers to health and wellbeing. When governments invest in community midwives – hiring locally, engaging in two-way information sharing, or consulting with local women leaders on public health decisions for propagating important health messages for safe motherhood both online and offline – they may not see immediate operational or public health wins. But in the long-term, they will build community trust and mutual respect, which can lead their communities to reciprocate, supporting public health, and their ongoing success. Now, more than ever, having midwives equipped with the expertise and tools to carry their vital work forward in online spaces will help them better support pregnant women navigating a complex information environment and continue building trust in healthcare voices like theirs. It will also contribute towards quality healthcare provision, the overall goals of public health and global health goals, and critically, towards leaving no one behind, the key subgoal of Universal Health Coverage for sustainable development.

## Data Availability

Data sharing is not applicable to this article as no new data were created.
